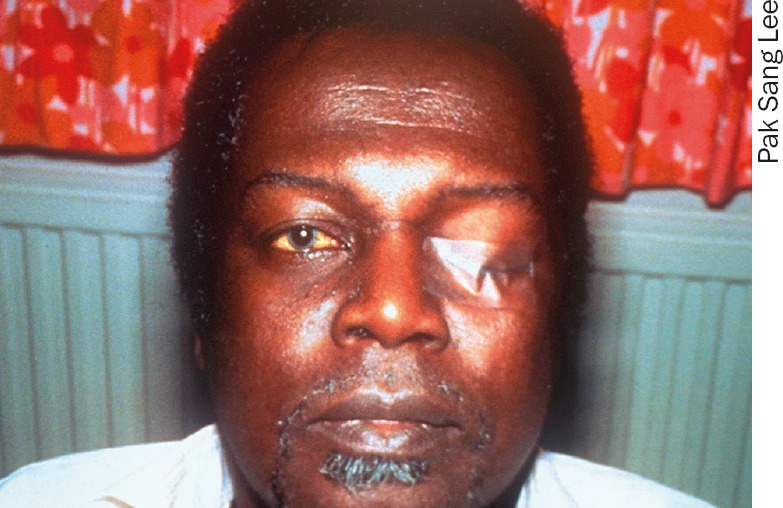# Taping an eyelid closed

**Published:** 2012

**Authors:** Sue Stevens

**Affiliations:** Former Nurse Advisor, Community Eye Health Journal, International Centre for Eye Health

**Figure:**
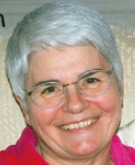
Sue Stevens

## Before performing any eye procedure

- Wash your hands (and afterwards too).
- Position the patient comfortably with head supported.
- Avoid anything that may distract you or the patient.
- Ensure good lighting.
- Always explain to the patient what you are going to do.

## Reasons for taping an eyelid closed

- To protect an eye with an anaesthetised cornea.
- To avoid exposure keratitis, e.g., when normal eyelid closure cannot be achieved.
  •To aid healing of an epithelial defect.
- To assist eyelid closure under an eye pad.

## You will need

- Scissors
- Waterproof adhesive tape: 2.5 centimetres (1 inch) wide

**NOTE**: Use only lightweight tape, as others are likely to cause a reaction when used on the sensitive skin of the eyelids.

## Preparation

- Carefully explain the procedure to the patient so she or he understands what will happen. It can be very alarming to find that your eye cannot open!
- Ensure the eyelid skin is clean and dry.
- Ask the patient to close both eyes.

## Method

**Figure 1.**
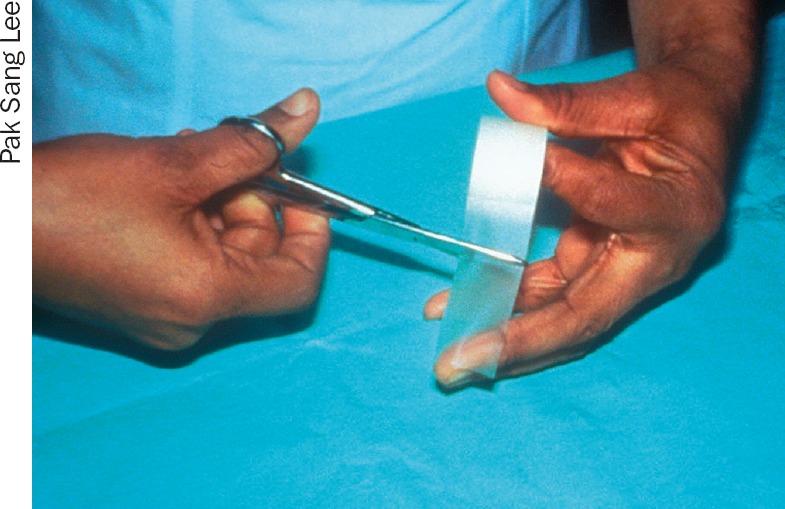


**Figure 2.**
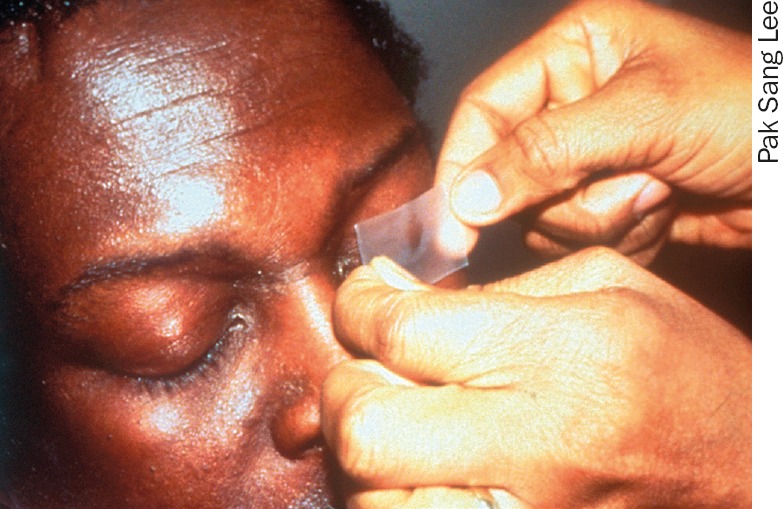


**Figure 3.**
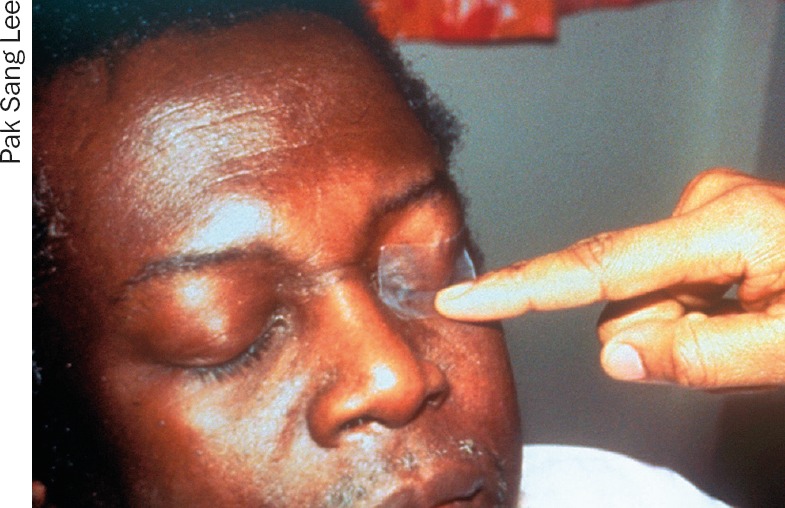


- Cut a piece of tape approximately 4 cm long (Figure 1).
- Hold the tape horizontally. Apply the top half of the tape to the lower half of the eyelid (Figure 2).
- Secure the bottom half of the tape to the skin below the lower eyelid (Figure 3).
- Check that closure is effective by asking the patient to open both eyes (Figure 4); this should be impossible for the taped eye.
- Reassure the patient again by reminding her or him of the aim of the procedure.

**NOTE**: The tape can become loose over time, so replace as necessary.

**Figure 4.**