# Supplementary material for: Taping an eyelid closed
Source: Community Eye Health. 2012;25(78):36. (PMC3483612)
Supplement: Related texts

# Brief report: Tape correction for lower eyelid entropion

**Published:** 2012

## Before performing any eye procedure

- Wash your hands (and afterwards too).
- Position the patient comfortably with head supported.
- Avoid anything that may distract you or the patient.
- Ensure good lighting.
- Always explain to the patient what you are going to do.

## Reasons for this procedure

Entropion is the turning in of the edges of the eyelid (usually the lower eyelid) so that the lashes rub against the eye surface. This procedure provides temporary correction of lower lid entropion in order to:

- relieve discomfort
- avoid corneal abrasion caused by the interned lashes (trichiasis).

## You will need

- Scissors
- Mirror
- Waterproof adhesive tape: 2.5 centimetres (1 inch) wide

**Figure 1.** 

**Figure 2.** 

**NOTE**: Use only lightweight tape, as others are likely to cause a reaction when used on the sensitive skin of the eyelids.

## Preparation

Sit or stand the patient comfortably and offer the mirror as a teaching aid.

## Method

- Cut a piece of tape about 3 cm (approximately 1.2 inches) in length.
- Gently evert (turn out) the lower eyelid to its former, normal position.
- Apply one end of the tape to the skin about half a centimetre (5 mm or about 0.2 inches) below the centre of the lower eyelid.
- Pull gently downwards on the tape, creating a horizontal fold in the skin below the eyelid (Figure 1).
- Secure the remainder of the tape to the facial skin, maintaining the skin fold (Figure 2). This should correct the lower eyelid position.
- Ask the patient to close, and then open, both eyes naturally. This will indicate whether the taping is effective and secure enough. It should still allow the eye to close completely.

**REMEMBER!**

Patients will have to learn to do this themselves. Allow them to practice a few times under your supervision. Remind them how important it is to replace the tape regularly in order to maintain the benefit. Ensure the patient has a supply of tape. Remind the patient to check that the taped eye can still close completely, as they could otherwise be at risk of exposure keratitis.
